# Movement-related EEG signatures associated with freezing of gait in Parkinson’s disease: an integrative analysis

**DOI:** 10.1093/braincomms/fcab277

**Published:** 2021-11-24

**Authors:** Fatemeh Karimi, Jiansheng Niu, Kim Gouweleeuw, Quincy Almeida, Ning Jiang

**Affiliations:** 1 Systems Design Engineering Department, University of Waterloo, Waterloo N2L 3G1, Canada; 2 Department of Human Media Interaction, University of Twente, 7522 NB Enschede, The Netherlands; 3 Department of Kinesiology and Physical Education, Wilfrid Laurier University, Waterloo N2L 3C5, Canada

**Keywords:** Parkinson’s disease, freezing of gait, supplementary motor area, movement-related cortical potential, brain oscillations

## Abstract

Freezing of gait is the most severe gait deficit associated with Parkinson’s disease and significantly affects patients’ independence and consequently their quality of life. The lack of a clear understanding of its underlying neurophysiological mechanism has resulted in limited effectiveness of the current treatment options. In this study, we investigated EEG features over (pre-)supplementary motor area and primary motor cortex during a simple cue-based ankle dorsiflexion movement. These features include movement-related cortical potentials (0.05–5 Hz) and brain oscillations (1–50 Hz). Electromyogram signal from the tibialis anterior muscle of the dominant foot was used to determine the movement onset. The EEG features before, during and following the onset of the movement were compared among three groups of participants: patients with freezing (*N* = 14, 11 males), patients without freezing (*N* = 14, 13 males) and healthy age-matched controls (*N* = 13, 10 males) with 15 recorded trials for each individual. Additionally, Parkinson’s disease patients with freezing of gait were separated into mild (*N* = 7) and severe cases (*N* = 5), so that EEG features associated with freezing severity could be investigated. The results indicated significant differences between patients with severe freezing of gait compared to healthy controls and patients without freezing of gait. In addition, patients with mild and severe freezing represented cortical activity differences. For patients with freezing, the initial component of movement-related cortical potential is significantly lower than that of the healthy controls (*P* = 0.002) and is affected by the severity of freezing. Furthermore, a striking absence of beta frequency band (12–35 Hz) desynchronization was observed in patients with freezing, especially low-beta frequency band over Cz, before the movement, which was also associated with the severity of the freezing of gait. Low-beta (13–20 Hz) and high-beta (21–35 Hz) frequency band activities represented unique features for each group. Beta event-related desynchronization over Cz present in healthy controls prior to movement onset, was partially replaced by the theta band (4–8 Hz) synchrony in patients with freezing. Patients with severe freezing also represented some level of theta band synchronization over contralateral supplementary motor area. This suggests the involvement of cognitive processing over the motor cortex in controlling cue-based voluntary movement as a compensatory mechanism associated with freezing of gait. The EEG features identified in this study are indicative of important freezing of gait clinical characteristics such as severity and contribute to a better understanding of the underlying neurophysiology of the mysterious phenomenon of freezing of gait.

## Teaser

Karimi et al. report that the severity of freezing of gait is associated with features of movement-related cortical potentials over the primary motor cortex and supplementary motor area. They also report that low- and high-beta activities and the presence of theta band synchronizations before movement execution are brain oscillations affected by freezing of gait severity.

## Graphical Abstract

**Graphical Abstract:**
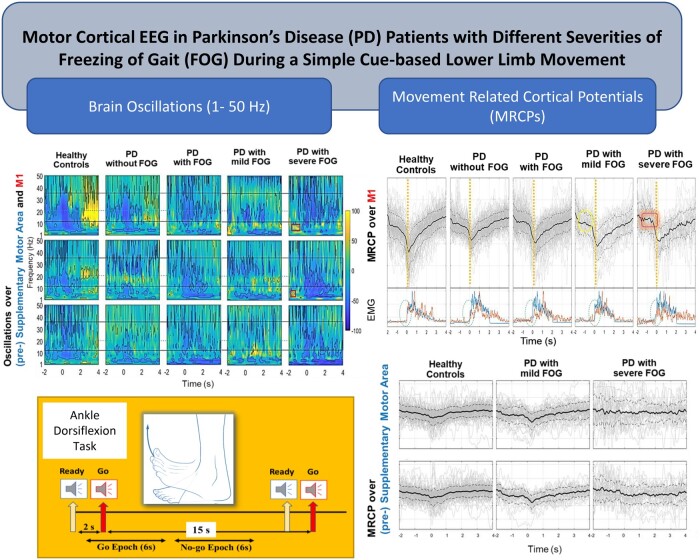

## Introduction

Freezing of gait (FOG) is a complex and disabling symptom in Parkinson’s disease, defined as a transient and sudden episode of inability to produce effective stepping despite the intention of gait.^1^ While dopaminergic treatments reduce the frequency and number of FOG episodes in most patients, the effectiveness of these treatment options is limited in Parkinson’s disease patients with FOG, especially in severe cases, making it a challenging healthcare problem in Parkinson’s disease.^2–6^ Although various methodological approaches have offered some insights, the underlying mechanism of FOG is still poorly understood due to its complexity and paroxysmal nature.^2^^,^^7^^,^^8^ More recently, the focus of Parkinson’s disease research has moved towards investigating dysfunction of all networks that involve basal ganglia (BG), including cerebellum and cortex, rather than focusing solely on the BG itself.^9^ For over-learned skills such as walking, most aspects of motor function are controlled at the cortical level, and the BG’s involvement is largely restricted to the regulation of movement gain.^10^ At the cortical level, the pre-supplementary motor area (SMA) along with SMA [(pre-)SMA], and primary motor (M1) cortex are some of the main components of the locomotor network involving direct and indirect pathways between the BG and the cerebral cortex, which contribute to movement planning and execution.^7^ Therefore, measuring activities of these areas prior, during and following lower limb movements in Parkinson’s disease patients without FOG along with Parkinson’s disease patients with different severities of FOG, may reveal abnormalities underlying FOG.

Movement-related cortical potential (MRCP) is a type of EEG modality, which starts approximately 1.5–2 s prior to a voluntary movement onset, with the frequency band and amplitude of 0–5 Hz and 5–30 µv, respectively. Considering the prominence of BG thalamocortical projections onto (pre-)SMA, alternations in different components of MRCP might be associated with features of FOG. Time course and amplitude of MRCP sub-components, including Bereitschaftspotential (BP) or contingent negative variation (CNV), in case of externally cued movements, motor potential (MP) and movement-monitoring potential (MMP) can be influenced by psychological status and the characteristics of the movement, such as level of movement intention, speed, precision and movement repetition.^11–13^ NS1 [negative slope of early BP (BP1)] and NS2 [steeper negative slope of late BP (BP2)] are two components of BP that precede the movement onset and reflect the activation of (pre-)SMA and M1, respectively.^11^^,^^12^^,^^14–16^ Various studies have investigated MRCP in the healthy population and in Parkinson’s disease, mostly during upper limb movements and some lower limb movements.^17^ However, discrepancies in changes in NS1 and NS2 still exist under different interventions. ^15^^,^^16^^,^^18–22^ While increased NS1 was reported in Parkinson’s disease patients after dopaminergic treatments; it does not influence NS2 by short-term dopaminergic treatment options.^23^ On the other hand, chronic administration of L-dopa results in increased NS2 rather than NS1.^24^ NS1 was found to increase with neurofeedback treatment in both Parkinson’s disease patients and healthy controls, while increased NS2 was achieved after pallidotomy in Parkinson’s disease patients.^25^^,^^26^ More detailed comparisons between treatment results on BP and CNV were found by using deep brain stimulation (DBS). CNV was increased during DBS; however, no difference was shown in BP.^27^^,^^28^ Most studies on BP in Parkinson’s disease involve the upper-limb movements, and, to the best knowledge of the authors, there is no research which investigates FOG with lower-limb MRCP. BP from Parkinson’s disease patients off medication was compared with controls during gait initiation and foot movement in a sitting position.^17^ Higher BP amplitude was reported when initiating a gait than moving a foot in a seated position in healthy controls, while BP amplitude in Parkinson’s disease does not show such a difference. The same experiment was replicated by another study to explore the relationship between BP and gait initiation failure (GIF), which is similar to the FOG symptom in Parkinson’s disease.^29^ A decreased BP amplitude was reported in GIF patients but not with Parkinson’s disease patients, which was regarded as an important piece of evidence to the different underlying mechanisms of GIF and Parkinson’s disease.

Event-related (de)synchronization (ERD/ERS) of different brain oscillations are other EEG features related to movement planning and execution.^30^ Beta frequency band (12–35 Hz) activities have been linked to motor function at the cortical level as well as in the subthalamic nucleus (STN) and globus pallidus interna.^31^ Beta band ERD/ERS has been widely investigated in Parkinson’s disease both at cortical and subcortical levels, and increased beta activities over the motor cortex were reported in numerous studies on Parkinson’s disease.^32–36^ Beta synchronizations in the STN lag beta activities in frontal and motor cortical areas with stable but different time delays in different cortical areas in Parkinson’s disease.^32^ This time delay is also different for the low and high-beta frequency bands. In recent studies with Parkinson’s disease patients with FOG and akinetic-rigid motor symptoms, abnormal dopamine resistant high-beta band (21–35 Hz) activities were reported in the STN, suggesting a relationship between FOG and beta frequency sub-bands.^32^^,^^37^ Maximal coherence in the high-beta activity was located in the midline cortex corresponding to the SMA, cingulate cortex and leg area of M1. In contrast, low-beta coherence was highest in the lateral M1 region.^37^ Increased beta frequency activities, especially high-beta frequency range, in the STN is associated with the onset of FOG episodes.^38^ In addition to abnormal beta band activities in Parkinson’s disease, excessive theta band (4–8 Hz) activities, which are associated with cognitive functions, were reported in central and frontal leads during FOG episodes, which, in turn, are associated with coupling between the pre-SMA and dorsal anterior cingulate.^36^ Brain dynamics alternations before and during FOG episodes have been explored in a number of cortical studies that use EEG to identify neural biomarkers for detection and prediction of freezing episodes.^39^

FOG as a complex multi-system lower body symptom in Parkinson’s disease requires a deeper understanding of the underlying mechanisms of this phenomenon both at the cortical and subcortical level. New techniques and advancements in EEG, which is a non-invasive portable technology, can be used for identification of reliable biomarkers. Understanding the abnormal cerebral activities related to FOG not only provides insights into the underlying mechanism of FOG and possibly more medication-based treatments and therapeutic interventions such as adaptive closed-loop DBS but may also offer recently emerged treatment or rehabilitation options based on a brain–computer interface (BCI), transcranial magnetic stimulation (TMS) and transcranial direct current stimulation (tDCS). In this study, multiple EEG features of (pre-)SMA and M1 are investigated in Parkinson’s disease with and without FOG, as well as for different severities of FOG, which are compared with healthy controls during a simple cued lower limb motor execution task.

## Materials and methods

### Participants

This study involved 14 Parkinson’s disease patients without FOG, 14 Parkinson’s disease patients with FOG and 13 age-matched healthy participants (Parkinson’s disease without FOG: mean age = 77 years, range = 65–87 years, 3 females; Parkinson’s disease with FOG: mean age = 74 years, range = 63–90 years, 1 female; controls: mean age = 77 years, range = 68–89 years, 3 females). The Parkinson’s disease patients were recruited from the Movement Disorders Research and Rehabilitation Center (MDRC) at the Wilfrid Laurier University (Waterloo, Ontario). Participants with any head trauma, neurological disorder, serious vision or hearing problems and severe movement control limitation such as dyskinesia were excluded. All patients were in their optimally medicated state to avoid the confound of exacerbated motor symptoms. Patients were assessed based on the Unified Parkinson’s Disease Rating Scale (UPDRS).

Parkinson’s disease patients with FOG were identified by the answer to question 14 in MDS-UPDRS-III (motor subsection), which confirms the presence of FOG. In addition, an experienced clinician reconfirmed the occurrence of FOG before each experiment session.^40^ The procedure involved a modified Timed Up and Go test where the participant would have started from a seated position, raised themselves out of a chair with arms across their chest, walked ∼3 m but through a door way into an adjacent clinic room that was cluttered with other desks and chairs, then returned to in front of their chair where they completed degree turns in both the left and right directions, before sitting back down. The goal of the study was to investigate the motor cortical abnormalities associated with FOG and its severity on (pre-)SMA and M1. For this purpose, various features of MRCP and brain oscillations of Parkinson’s disease with different degrees of FOG are compared with healthy controls as well as Parkinson’s disease without FOG.

Parkinson’s disease patients with FOG were divided into two subgroups of Parkinson’s disease with mild and severe FOG. Severe freezers were defined as those who experienced observable FOG episodes whenever walking or turning that severely affect their daily activities and independence, while mild freezers were defined as those who experienced FOG occasionally when provoked only during more complex tasks such as turning (based on patient history). In addition, the participants were instructed to perform 20 trials of videotaped walking tasks on a 10-m walkway. Participants were asked to walk after hearing an auditory ‘go’ cue. Parkinson’s disease with FOG who experienced FOG episodes longer than 3 s during turning or normal walking were considered Parkinson’s disease with severe FOG. The videotaped walking tasks were used to determine the dominant foot for each participant.

Healthy participants were recruited from The Waterloo Research in Aging Participant pool at the University of Waterloo. The sample size was determined by availability of Parkinson’s disease patients. The study was approved by the Research Ethics Board at the University of Waterloo and Wilfrid Laurier University. A written informed consent form was obtained from each participant prior to the experiment, according to the Declaration of Helsinki.

### EEG and EMG recordings

EEG data were recorded using a 32-channel wireless EEG system (g.Nautilus, Guger Technologies, Austria). EEG signals were sampled at a sampling rate of 250 Hz. EEG data were collected from 17 channels following 10–20 international standard positions: FP1, FP2, AF3, AF4, F3, Fz, F4, FC1, FC2, C3, Cz, C4, CP1, CP2, P3, Pz and P4. The reference electrode was placed on the right ear lobe.

For all individuals, the EMG was acquired using an 8-channel TELEMYO 2400 system (NORAXON INC). Four wireless EMG sensors with a sampling frequency of 1000 Hz were placed on the tibialis anterior (TA) and soleus muscles (SOL) on both legs.

### Experimental procedures

All participants were invited to the MDRC for the experimental sessions. For Parkinson’s disease patients, the respective clinical assessment (which included UPDRS-III to confirm motor symptom severity) was performed within 2 weeks of the experimental session. During the experiment, participants were instructed to perform ankle dorsiflexion (ADF) (e.g. lifting the toe) at their normal pace to the maximum possible contraction with the dominant foot while sitting in a comfortable chair with their arms rested in armrests. The participants were asked to release their toes after reaching the maximum contraction. To minimize eyes or head movements and reduce the cognitive load unrelated to the cues, they were asked to look at the center of a black ‘+’ sign on a white background. One session with 15 trials was recorded for each participant, with an interval of 15 s between every 2 trials. Participants were expected to prepare for the task when they heard ‘ready’ and execute ADF when they heard the ‘go’ cue. The ‘ready’ and ‘go’ auditory cues, with a 2 s interval, were played for each trial through a speaker with a computer-generated voice. In this study, ‘Go’ epochs, which contain the signal from 2 s before the movement onset to 4 s after the onset, were analysed to evaluate the cortical activities during movement preparation and execution (Supplementary Fig. 1 represents the time course of the auditory cues and extracted ‘Go’ epochs).

### Data processing

EEG and EMG data were analysed offline after the experiment session using a customized Matlab function (Mathworks, USA R2020a). EMG signals recorded from the TA muscles of the dominant foot (EMG -TA) were used to identify onset timings of the ADF. EMG-TA was initially filtered using a second-order Butterworth band-pass filter with the bandwidth between 20 Hz and 120 Hz, then down-sampled to 250 Hz to maintain consistency with the EEG data. To enhance the detection accuracy of the movement onset, the Teager–Kaiser Energy Operator (TKEO) was applied to the EMG data.^41^ Finally, a threshold value was manually selected for each subject to determine the movement onset. The onset of the muscle activity was identified visually as the point in time where the amplitude of the EMG signal after TKEO clearly increased from baseline after the auditory ‘go’ cue.

For EEG processing, two different pre-processing paths were performed to analyse MRCP and brain oscillations levels. To analyse the MRCP, the EEG data were initially band-pass filtered by a third-order Butterworth filter between 0.05 Hz and 5 Hz. In order to avoid phase distortions, zero-phase forward and backward filtering procedure was used. The filtered EEG data were processed by the extended infomax independent component analysis (ICA) algorithm using the *EEGLAB* function ‘*runica.m*’ (MATLAB, CA, USA). Source components containing eye blinks, severe head motion or EMG artifacts were manually removed by visual inspections of the scalp topographies and waveforms.^42^ In order to minimize bias, two independent experts identified artifactual components, and only components that were identified by both experts were removed. Lastly, trials with the peak amplitude of negativity on Cz channel outside the range of [−1.5 to 2] s with respect to movement onset were removed as outliers.

For brain oscillations, similar to MRCP, EEG was first filtered by a third-order Butterworth filter between 0.05 Hz and 50 Hz, followed by ICA to remove artifactual components. For generating ERD/ERS time–frequency representation, small Laplacian filters were applied on Cz, Fc1 and Fc2 to reduce the effect of volume conduction.^43^ Laplacian spatial filter was implemented by subtracting the averaged signal of the four surrounding orthogonal electrodes from the centre electrode. The time–frequency representations of the power were computed using Morlet wavelets with five cycles between 1 Hz and 50 Hz, and ERD was calculated with a baseline from −4 s to −2 s.^43^^,^^44^ The outliers were discarded from further analysis based on the excessive muscle activities in both leg muscles before the auditory ‘go’ cue, EMG activity in the opposite leg during the motor task, and head motion detected by Inertial Measurement Unit mounted on participant’s neck. A total number of 122 ± 2 trials in each group was used for oscillatory analysis. For participants whose left leg was dominant, the EEG channels on the left and right sides were switched during the analysis.

### MRCP features

Five features of MRCP over M1 (Cz channel) were studied on a single trial basis: (i) peak amplitude of negativity; (ii) time course of the peak negativity; (iii) NS1; (iv) NS2; and (v) MRCP rebound rate.^45^ EMG features include peak amplitude of the EMG-TA, the delay between peak amplitude negativity of MRCP, and peak of EMG-TA. In each trial, the peak amplitude of negativity from MRCP was defined as the lowest value between −1 s and 1.5 s with respect to the movement onset. NS1 was calculated as the difference between the amplitude of the signal 1.4 s before the peak negativity and the amplitude computed 0.4 s before peak negativity, divided by 1 s. NS2 was calculated as the difference between the amplitude of the signal 400 ms before the peak negativity and the amplitude of peak negativity, divided by 0.4 s. The rebound rate of the MRCP was calculated as the difference between the amplitude of the potential at 1.5 s after the peak negativity and the peak amplitude of negativity, divided by 1.5 s.^45^

### Statistical analysis

Demographic and clinical characteristics were analysed using independent samples *t*-tests and one-way ANOVA. To investigate the effect of FOG and its severity on features of MRCP, one-way ANOVA with a significance level of 0.05 was carried out to compare MRCP features across participant groups. To assess main effect of groups, ANOVA with three levels of group factors: Healthy, Parkinson’s disease without FOG and Parkinson’s disease with FOG were conducted on the data. To compare the effect of FOG severity on MRCP features, the group factor included: healthy, Parkinson’s disease with mild FOG and Parkinson’s disease with severe FOG. When a significant main effect was found, Tukey’s test was used as a *posthoc* test.

Regarding the time–frequency representations of ERD/ERS with significant areas, significant time–frequency areas were calculated using the bootstrap re-sampling method for each group. A permutation test was applied to calculate the significant time–frequency areas when analysing the difference between groups.^46^^,^^47^ All statistical analysis was performed in MATLAB 2020 a.

### Data availability

All derived and anonymized individual data are available at http://ieee-dataport.org/documents/fog-severity-eegemg.^48^

## Results

Demographic and clinical characteristics are presented in Table 1. Healthy controls, Parkinson’s disease without FOG and Parkinson’s disease with FOG groups were matched for age [*F* (2,39) = 1.1, *P* = 0.3]. Parkinson’s disease without FOG and Parkinson’s disease with FOG were also matched for UPDRS, levodopa equivalent dose (LED) and disease duration (*P* = 0.4, *P* = 0.2 and *P* = 0.4, respectively). Also, Parkinson’s disease with mild and severe FOG groups did not differ in age (*P* = 0.4), severity of motor symptoms (UPDRS-III: *P* = 0.4), LED (*P* = 0.07), disease duration (*P* = 0.3). Individual participant details of all participants are presented in Supplementary Table 1.

**Table 1:** Mean ± standard deviations for participant demographics and clinical characteristics

	HC	Parkinson’s disease **without FOG**	Parkinson’s disease **with FOG**	Parkinson’s disease **with mild FOG**	Parkinson’s disease **with severe FOG**
*N* (male/female)	13 (10/3)	14 (11/3)	14 (13/1)	7 (6/1)	5 (5/0)
Age (year)	77.61 ± 5.65	74.5 ± 6.67	77.64 ± 7.41	76.7 ± 7.5	79.6 ± 1.14
Disease duration (year)	NA	8.07 ± 5.18	9.64 ± 5.49	11 ± 5.74	7.8 ± 5.11
UPDRS-III	NA	28.66 ± 7.16	31.65 ± 12.12	34.21 ± 12.7	28 ± 6.85
LED (mg/day)	NA	449.42 ± 358.59	639.71 ± 419.72	585.8 ± 265.7	969 ± 468.03

HC, healthy controls.

### MRCP features are associated with the severity of FOG

**Figure 1:**
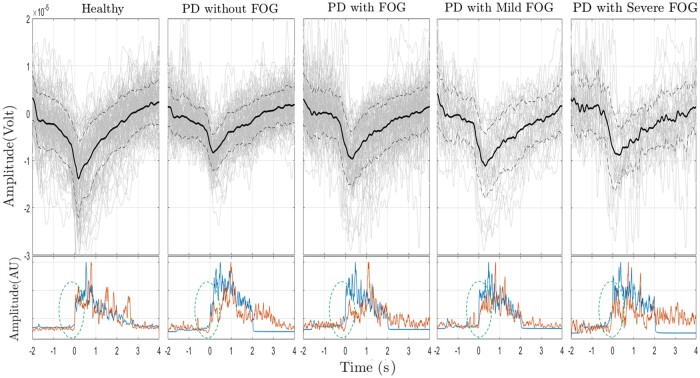
**Average MRCP over ‘Go’ epochs from Cz channel and normalized EMG signal from TA and SOL muscles. In each group, the top plots are the epoch averages of ‘Go’ epochs.** The thick solid line is the average over all trials, and thinner grey lines are the single trials for all subjects. The dashed lines indicate the average standard deviation in each case. In the lower row of each group, the average of the normalized EMG signal over all trials for the TA muscle (blue line) and SOL muscle (red line) are presented over the time course of the ‘Go’ epoch. Dashed green oval includes the time of the activity onset in TA and SOL muscles.

The average MRCP of ‘Go’ epochs and standard deviation of each group for the Cz channel with background single trials within each group are presented in Fig. 1. Normalized average EMG signals (with respect to EMG-TA) from the TA muscle (blue line) and SOL muscle (red line) over the ‘Go’ epoch are also shown in the lower row. Visual inspections show that the largest NS1 was observable in healthy controls and the lowest NS1 belonged to the Parkinson’s disease with FOG and especially Parkinson’s disease with severe FOG group. The amplitude of MRCP from Parkinson’s disease patients, especially Parkinson’s disease without FOG, was generally lower than healthy controls. However, it should be noted that the amplitude of the EMG signal from TA muscle seems to be consistent with MRCP amplitude (Supplementary Fig. 2 represents the relative MRCP and EMG amplitude for healthy, Parkinson’s disease without and with FOG). A time delay between the onset of TA and SOL muscle activation was also observable in the Parkinson’s disease with FOG group, which is visualized by the dashed green oval in Fig. 1. MRCP features for all groups are reported in Table 2. Comparison between healthy, Parkinson’s disease with and without FOG, results from the one-way ANOVA showed that the amplitude of peak negativity at Cz, the NS1, the amplitude of EMG-TA, the time of EMG-TA peak, and latency between MRCP peak and EMG peak were significantly different among groups [peak negativity at Cz: *F*(2, 284) = 22.2, *P* < 0.001, NS1: *F*(2, 284) = 5.8, *P* = 0.003, amplitude of EMG-TA: *F*(2, 284) = 16.2, *P* < 0.001, Time of the peak of EMG-TA: *F*(2, 284) =6.3, *P* = 0.002, latency between MRCP peak and EMG peak: *F*(2, 284) = 4.8, *P* = 0.008]. The multiple comparison tests indicated that the peak negativity over Cz in Parkinson’s disease without FOG is significantly lower than healthy controls and Parkinson’s disease with FOG (*P* = 0.01 and *P* = 0.03, respectively). The comparison tests also showed that the NS1 from Parkinson’s disease with FOG was significantly lower than the NS1 from the healthy controls (*P* = 0.002). Amplitude of EMG-TA from healthy participants were significantly higher than the Parkinson’s disease with and without FOG (*P* < 00.1). The time of the EMG peak from healthy and Parkinson’s disease without FOG was also significantly different (*P* = 0.001). Latency between MRCP peak and EMG peak of Parkinson’s disease without FOG was significantly higher than healthy controls and Parkinson’s disease with FOG (*P* = 0.01 and *P* = 0.03, respectively). In contrast, NS2, rebound rate and the time of the peak negativity over Cz were not significantly different across the three groups: healthy, Parkinson’s disease with FOG and Parkinson’s disease without FOG.

**Table 2:** Mean ± standard deviations of MRCP features over Cz, significant test statistics are marked with ‘^*^’, ‘^**^’, ‘^***^’, ‘^****^’, ‘^*****^’ for different group effects

	Three main groups			Parkinson’s disease **with FOG**	
MRCP features	HC	Parkinson’s disease **without FOG**	Parkinson’s disease **with FOG**	Mild FOG	Severe FOG
Peak amplitude of negativity over Cz	−16.1 ± 7.4	−10.5 ± 3.8 ^*^	−12.6 ± 5.7	−13.6 ± 6.2	−13.7 ± 6.1
Peak amplitude of EMG-TA	946.5 ± 274.2	737.3 ± 319.3^**^	691.6 ± 399.6^***^	825.5 ± 401.7	512 ± 221.9^*****^
Latency between MRCP peak and EMG-TA peak	−22.7 ± 110.5	−67.8 ± 121.5^*^	−28.4 ± 92.3	−13.2 ± 97.7	−35.6 ± 109
Time of the peak negativity over Cz	338.9 ± 87.1	335.8 ± 93.6	349.2 ± 89.4	358.3 ± 82.2	360.1 ± 120.1
Time of the peak of EMG-TA	361.7 ± 80.2	403.7 ± 95.8^**^	377.6 ± 71.03	371.6 ± 57	395.8 ± 93
NS1 of Cz	−3 ± 5.3	−1.7 ± 3.7	−0.8 ± 4.7^***^	−1 ± 4	−0.6 ± 6.3^****^
NS2 of Cz	−12.8 ± 11.3	−10.5 ± 0.9	−14.4 ± 14.9	−13.2 ± 12	−18.6 ± 21.2
Rebound rate Cz	3.4 ± 4.6	2.8 ± 3.3	2 ± 4.8	1.2 ± 4.7	2.5 ± 6.2

* *P* < 0.05 Parkinson’s disease *without FOG* versus HC and Parkinson’s disease *with FOG*. ^**^P < 0.05 Parkinson’s disease *without FOG* versus HC.

˚^***^ *P* < 0.05 Parkinson’s disease *with FOG* versus HC.

^****^ *P* < 0.05 Parkinson’s disease with severe FOG versus HC.

^*****^ *P* < 0.05 Parkinson’s disease with severe FOG versus HC and Parkinson’s disease with mild FOG.

**Table 3:** Coefficient of variation for three channels over M1 and (pre-)SMA

	Three main groups			Parkinson’s disease **with FOG**	
Coefficient of variation of different channels	HC	Parkinson’s disease **without FOG**	Parkinson’s disease **with FOG**	Mild FOG	Severe FOG
**FC1**	3.9	5.3	4.8	2.5	12.6
**FC2**	5.3	5.3	3.9	2.1	7.1
**Cz**	1.9	2.5	2.6	2.0	3.2

**Figure 2:**
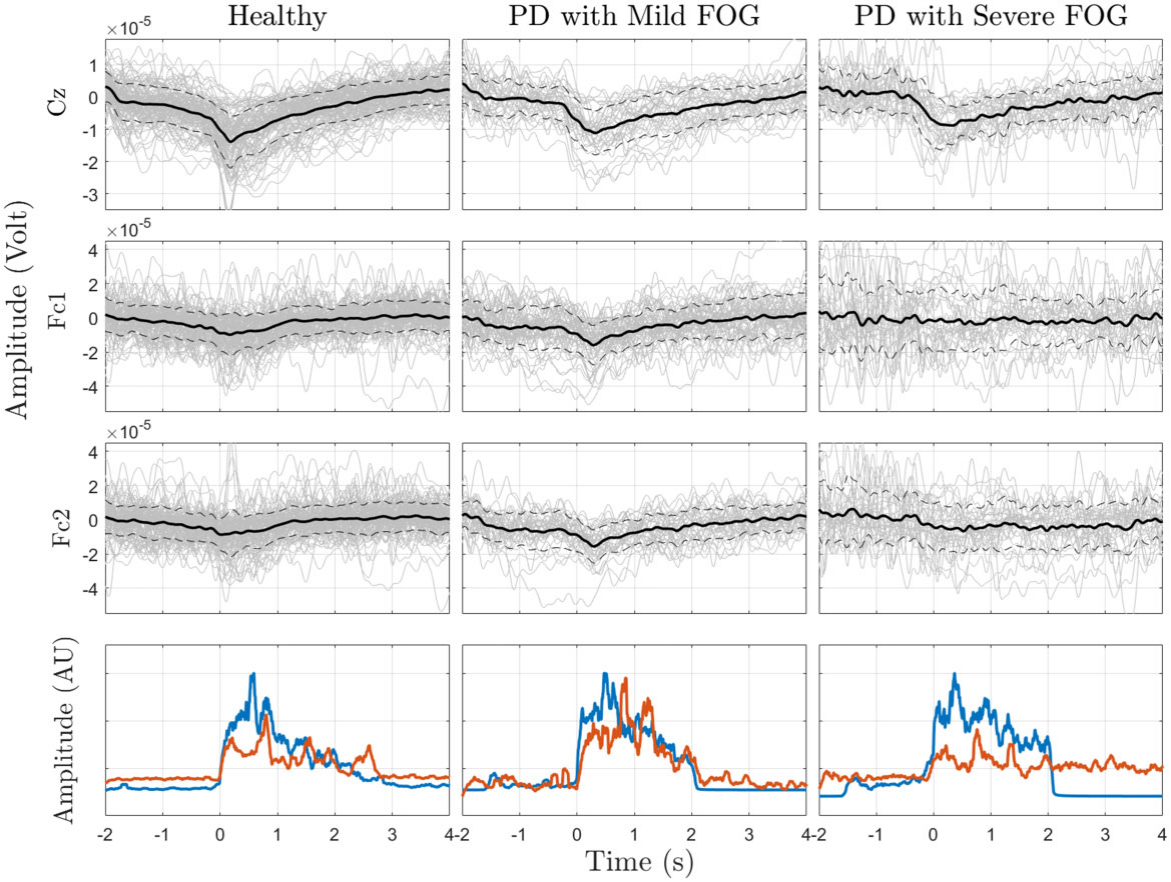
**Average MRCP over ‘Go’ epochs from Cz, Fc1, and Fc2 channels and normalized EMG from TA and SOL muscles for healthy controls, and Parkinson’s disease with mild and severe FOG. In each group, the top plots are the MRCP averages of ‘Go’ epochs.** The thick solid line is the average over all trials, and the other thinner grey lines are the single trials for all subjects. In the lower row of each group, the average of EMG signal over all trials for TA muscle (blue line) and SOL muscle (red line), respectively. The dashed lines indicate the average standard deviation in each case.

One-way ANOVA for features of MRCP over Cz for healthy participants, Parkinson’s disease with mild and severe FOG found significant differences for NS1 and amplitude of EMG-TA [*F* (2, 166)=3.65, *P* = 0.02]; [*F* (2, 166) = 25.1, *P* < 0.001], respectively. The multiple comparisons showed that NS1 from the Parkinson’s disease with severe FOG group was significantly lower than the healthy group (*P* = 0.02). Peak amplitude of EMG-TA from Parkinson’s disease with severe FOG was significantly lower than both healthy and Parkinson’s disease with mild FOG (*P* < 0.001). In contrast, NS2 and rebound rate were not significantly different across healthy, mild FOG and severe FOG. Although the time course of peak negativity was not significantly different among groups, direct observations show that the standard deviation (SD) of Parkinson’s disease patients with severe FOG was higher among the three groups resulting in a flatter peak negativity for this group. Direct observations also show that inconsistency between the onset of EMG activities from the TA and SOL muscles were present in Parkinson’s disease patients with severe FOG, while absent in the other groups (the onset of EMG activities is marked with green dashed ovals). In Fig. 2, the average MRCPs are presented for healthy, Parkinson’s disease with mild and severe FOG for three channels: Cz, FC1 and FC2. The most prominent difference between Parkinson’s disease with severe FOG and healthy as well as Parkinson’s disease with mild FOG in MRCP frequency range, was that the averaged amplitude of the signal over (pre-)SMA (Fc1 and Fc2) were lower in the Parkinson’s disease with severe FOG group compared to the two other groups. This difference could be a result of lower temporal consistency across trials in Parkinson’s disease with severe FOG in (pre-)SMA. Parkinson’s disease with severe FOG group showed less temporal consistency across trials especially over the contralateral channel (Fc1). To quantify the temporal variations of the signals over (pre-)SMA and M1, the coefficient of variation, the standard deviation divided by mean value, was calculated and presented in Table 2 for all groups. The results conform the higher temporal variation of the signals in Parkinson’s disease with severe FOG, particularly over (pre-)SMA compared to other groups.

**Figure 3:**
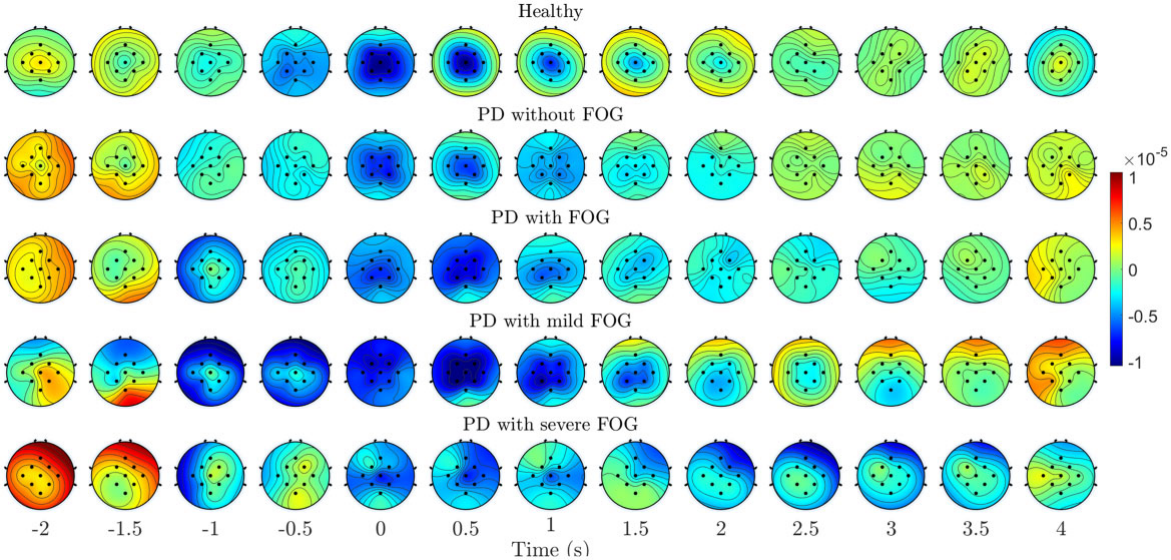
**Topographic maps of five groups.** Topographical plots of Healthy controls, Parkinson’s disease patients with FOG, Parkinson’s disease patients without FOG, Parkinson’s disease patients with mild FOG, Parkinson’s disease patients with severe FOG over MRCP frequency range. The voltages (Unit: Volt) of nine electrodes are represented as different colours in topographical maps. Different topographical maps along 13 time points between −2 s and 4 s with an interval of 0.5 s.

Figure 3 represents the spatiotemporal grand average of MRCP amplitude representation for all groups over ‘Go’ epochs from Cz. Despite healthy controls and Parkinson’s disease without FOG, Parkinson’s disease with different levels of FOG did not show activities over Cz at the time of the ’ready’ cue (*t* = −2 s). Additionally, Parkinson’s disease with severe FOG did not represent any localized activity over Cz before movement onset (*t* = 0 s), while Parkinson’s disease with mild represent localized activities over Cz during preparation period. Parkinson’s disease with severe FOG also represented overactive frontal and parietal cortices during movement.

### Theta, low-beta and high-beta frequency bands are associated with FOG

**Figure 4:**
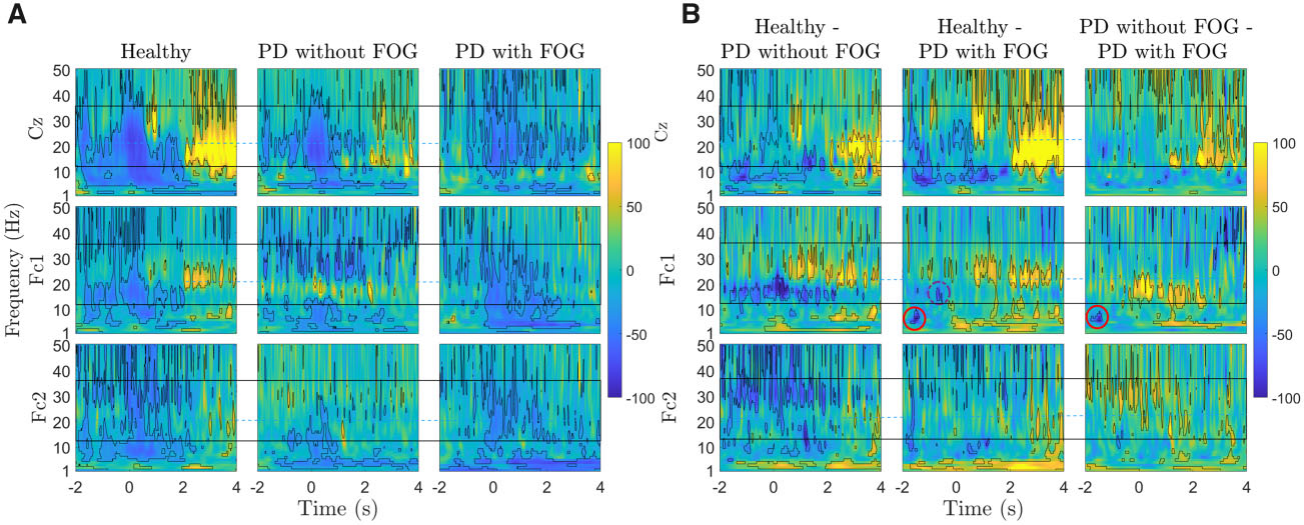
**Group differences in movement-related spectral power changes of healthy controls, and Parkinson’s disease without and with FOG.** Time–frequency representations of ERD/ERS and ERD/ERS differences in three channels (Cz, Fc1 and Fc2) of three groups: Healthy controls, Parkinson’s disease patients without FOG, and Parkinson’s disease patients with FOG. In plot A, ERD/ERS indicating percentage change relative to baseline of −4 s to −2 s are represented as blue/yellow colours, respectively, between 1 Hz and 50 Hz from −2 s to 4 s. significant areas are calculated with bootstrap re-sampling methods (*P* < 0.05) and outlined by black contours. In plot B, time–frequency representations of ERD/ERS differences in three channels (Cz, Fc1 and Fc2) among three groups, indicated as ‘Healthy—PD without FOG’, ‘Healthy—PD with FOG’, ‘PD without FOG—PD with FOG’. The solid black rectangle indicates the beta band frequency range, and the blue horizontal line separates low- and high**-**beta band frequency range (12–21 Hz) and (21–35 Hz), respectively. Red and purple dashed circles indicate theta and low**-**beta activity differences between Parkinson’s disease with FOG and other groups, respectively.

In Fig. 4, the time–frequency representations of the EEG signal between 1 Hz and 50 Hz of small Laplacian filtered Cz, Fc1 and Fc2 channels are presented for healthy controls, Parkinson’s disease with FOG and Parkinson’s disease without FOG (figures without permutation-corrected statistical significance are presented in Supplementary Fig. 3). Over Cz, healthy participants show continued low-beta band ERD before the onset of the movement as well as high-beta ERD at the time of the ‘ready’ cue and during movement execution. Parkinson’s disease patients without FOG presented a similar but lower low-beta and high-beta frequency band ERD pattern. In the Parkinson’s disease with FOG group, the low-beta ERD was highly suppressed and partially replaced by beta ERS during movement preparation and beta band ERD was interrupted during motor execution. In Parkinson’s disease patients with FOG, some levels of theta ERS were also present prior to the movement onset over both Cz and Fc1. In addition to pre-movement differences over Cz, beta ERS was present 2 s to 4 s after movement in healthy participants, while Parkinson’s disease patients did not show such ERS over the same time course, especially Parkinson’s disease with FOG.

In Fc1, which was basically the contralateral channel, the distinct and contrasting high-beta and low-beta band activities were observable across group. Low-beta ERD during movement preparation was completely missing in Parkinson’s disease with FOG. On the other hand, in Parkinson’s disease without FOG, continuous low-beta ERS was spanned over the whole-time course of the epoch and unlike other groups, no low-beta ERD was present over this channel, during movement execution. In both Parkinson’s disease groups, high-beta band ERD was observed during movement preparation and execution. This similarity might suggest a compensatory mechanism in Parkinson’s disease.

In Fc2, the ipsilateral channel, the low-beta ERD before the movement was more interrupted compared to Cz and Fc1 in healthy participants. In the healthy group, repetitive high-beta ERD before movement was missing in Parkinson’s disease groups. Figure 4B represents differences between every two groups. Healthy controls and Parkinson’s disease without FOG group did not show significant before movement in beta frequency band before movement. However, Parkinson’s disease without FOG represented delayed high-beta ERD over Cz at movement onset. The healthy group and Parkinson’s disease with FOG show significant differences in low-beta activities, especially before movement. Healthy and both Parkinson’s disease groups shared similar differences in the theta frequency band. Significant differences over Fc1 confirmed strikingly distinct high-beta and low-beta band activities when comparing groups. Low-beta frequency band represented main differences between Parkinson’s disease without FOG and other group. Theta ERS before movement onset in Parkinson’s disease with FOG represents significant difference between Parkinson’s disease with FOG with other groups (marked with a red solid circle). Additionally, a brief significantly different low-beta ERS is also observable before movement initiation in Parkinson’s disease with FOG compared with healthy controls (marked with a purple dashed circle). In Fc2, healthy and Parkinson’s disease with FOG share similarities, while high-beta activities represent the main difference between Parkinson’s disease without FOG with the other two groups.

**Figure 5:**
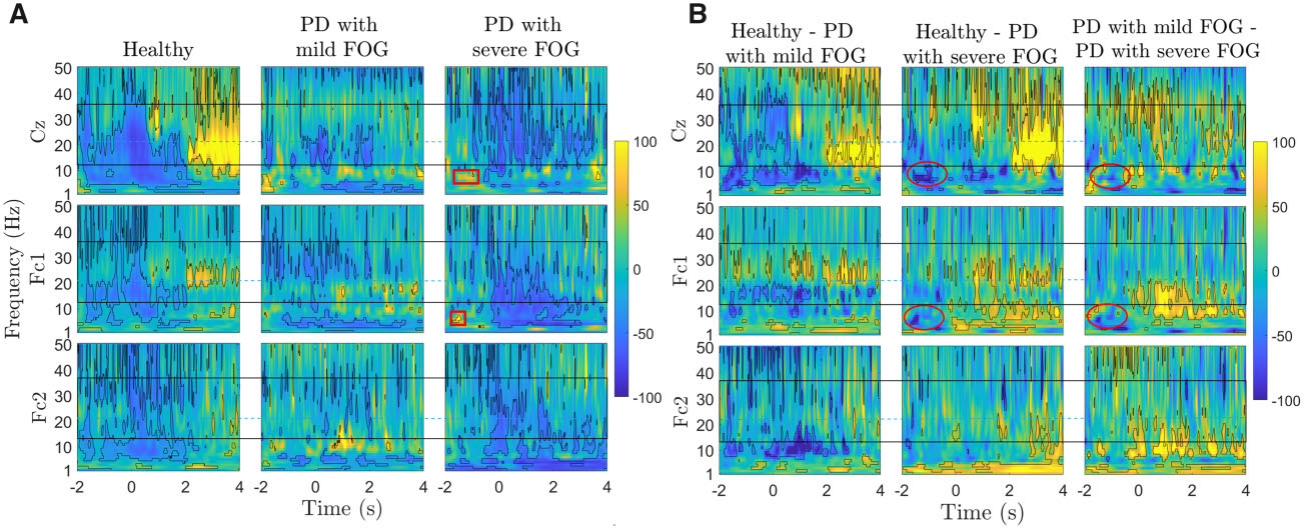
**Group differences in movement-related spectral power changes of healthy controls and Parkinson’s disease with different FOG severities.** Time–frequency representations of ERD/ERS in three channels (Cz, Fc1 and Fc2) of three groups: Healthy controls, Parkinson’s disease patients with mild FOG and Parkinson’s disease patients with severe FOG. In plot A, ERD**/**ERS indicating significant areas relative to a baseline of −4 s to −2 s are represented as blue/yellow colours, respectively, between 1 and 50 Hz from −2 s to 4 s. significant areas are calculated with bootstrap re-sampling methods (*P* < 0.05) and outlined by black contours. In plot B, time–frequency representations of ERD/ERS differences in three channels (Cz, Fc1 and Fc2) among three groups, indicated as ‘Healthy—PD with mild FOG’, ‘Healthy—PD with severe FOG’, ‘PD with mild FOG—PD with severe FOG’. Significant areas are calculated with a permutation test (*P* = 0.05) and outlined by black contours. The solid black rectangle indicates the beta band frequency range, and the blue horizontal line separates low- and high**-**beta band frequency range. Red ovals show theta band activities.

In Fig. 5, the time–frequency representations of the EEG signal between 1 Hz and 50 Hz on Cz, Fc1 and Fc2 are presented for healthy controls and FOG with different severities (figure without permutation-corrected statistical significance are presented in Supplementary Fig. 4). Theta and low-beta ERS before movement represent common frequency activity patterns for both FOG severities compared to healthy controls. Theta ERS represented a longer time course during movement preparation in Parkinson’s disease with severe FOG compared to Parkinson’s disease with mild FOG (theta ERS is marked with red rectangles). The high-beta frequent synchrony in mild FOG over Cz represents a unique feature of this group over Cz. In Fc1, similar to Parkinson’s disease without FOG, Parkinson’s disease with mild FOG showed excessive high-beta ERD before movement, continuous low-beta synchrony over the whole epoch, which might be related to tremor in Parkinson’s disease with mild FOG. Theta ERS over Fc1 during movement execution was a shared feature between Parkinson’s disease with mild and severe FOG groups.

Figure 5B represents the time–frequency differences across the healthy controls and two FOG subgroups over Cz, Fc1 and Fc2 (figure without permutation-corrected statistical significance are presented in Supplementary Fig. 4). A distinct high-beta and low-beta band were obvious when comparing the healthy group and Parkinson’s disease with mild FOG, especially over Fc1, which was similar to Parkinson’s disease without FOG. Lack of high-beta band ERD during movement was the other significant EEG feature for Parkinson’s disease with mild FOG over Cz. For Parkinson’s disease with severe FOG, theta band activities over Cz and Fc1 prior to the movement onset (marked with a red oval) along with excessive high-beta ERD over M1 represented the two main features of this group. Fc2, the contralateral channel, was the only channel that did not show any level of theta ERS in Parkinson’s disease with severe FOG. Parkinson’s disease with mild FOG also represented the unique feature of absent low-beta ERD during movement compared with healthy controls and Parkinson’s disease with severe FOG over Fc2.

## Discussion

The motor cortex is a pivotal brain structure involved in movement planning and execution through communication with the BG, cerebellum and spinal cord. Decreased dopamine level in Parkinson’s disease highly affects motor cortex functionality and circuitry, emphasizing the key role of cortical regions in the motor symptoms of the disease.^49^ In this study, a cue-based lower limb movement was used to investigate the motor cortical abnormalities associated with FOG and its severity during a simple lower limb motor execution task rather than FOG episodes. The results from this study showed that the motor cortical activities of Parkinson’s disease patients with FOG are not only different from healthy controls and Parkinson’s disease patients who do not experience FOG, but also Parkinson’s disease with mild and severe FOG show significant differences. This might suggest the intrinsic differences between FOG subtypes and severities. One of the most significant FOG-associated EEG abnormalities was the early component of MRCP. Parkinson’s disease with severe FOG had a significantly lower NS1 compared with healthy controls, and the slope of the NS1 decreased as the severity of FOG increased. NS1 originates from the pyramidal neurons activities in pre-SMA and cingulate motor area.^12^^,^^50^^,^^51^ (Pre-)SMA, at different layers, is engaged in motor learning, integrating and processing sequential elements, as well as planning and performing well-learned movements.^11^^,^^52–55^ To perform a movement, (pre-)SMA sends BP to the BG, and for each sequence of well-learned movements, a signal from BG is sent to SMA showing the termination of the sequence.^56^ Gait, as a well-learned movement sequence, critically depends on the functionality of (pre-)SMA. Impaired pre-SMA and loss of intra-cortically projecting pyramidal cells in this cortical region was reported in Parkinson’s disease.^57–60^ In most studies, investigation of BP and SMA is limited to Parkinson’s disease patients regardless of the type or severity of motor symptoms. The results from the NS1 differences in mild and severe freezers show the importance of further research on (pre-)SMA in patients with different FOG severities and subtypes.

Along with BP, the amplitude of MRCP was the other investigated EEG feature. The amplitude of the MRCP was lower for Parkinson’s disease without FOG group compared to controls but independent from FOG. It should be noted that the amplitude of the MRCP has been shown to be correlated with the amplitude of EMG.^61^ This suggests that the peak negativity of MRCP could be related to lower EMG amplitude, and not necessarily to FOG. Although the MRCP amplitude from Parkinson’s disease with FOG is slightly higher than Parkinson’s disease without FOG, they represent a lower peak of EMG signal among all groups (Table 2), which might be a result of time inconsistency of muscle activities in Parkinson’s disease patients with FOG. Analysis of the onset of EMG activities in both the TA and SOL muscle is beyond the scope of this paper; however, direct observations indicate that there is an inconsistency between the onset of EMG activities from the two muscles in Parkinson’s disease with FOG compared with other groups (green oval in Fig. 1). Flatter peak of averaged MRCP in severe FOG and inconsistent single-trial MRCPs over (pre-)SMA and EMG onsets in both foot muscles compared to mild FOG also show that FOG might be a result of a timing issue in the muscles.

Findings of this study also suggest distinct low- and high-beta band cortical abnormalities among Parkinson’s disease patients without FOG as well as for different levels of FOG severity during movement preparation and execution (Figs 4 and 5), which was consistent with the previous studies.^37^ Distinct low- and high-beta frequency bands shared remarkable similarities and differences across groups. Lack of low-beta ERD and partial ERS over Cz was a FOG-associated EEG signature common between both mild and severe FOG. While Parkinson’s disease with mild FOG represented low-beta ERD over Cz at the time of the ‘ready’ cue, Parkinson’s disease with severe FOG group did not represent any low-beta ERS before movement onset. Parkinson’s disease with mild FOG shares some similar high- and low-beta activities with Parkinson’s disease without FOG, especially over FC1. However, lack of low-beta ERD during movement execution over FC2 and frequent high-beta synchrony over Cz are two exclusive features of Parkinson’s disease with mild FOG. Considering the fact that Parkinson’s disease without FOG and Parkinson’s disease with mild FOG shows similar patterns over certain cortical regions and frequency bands, it can be suggested that mild FOG and severe FOG could be affected by different underlying FOG mechanisms. For instance, the high-beta ERD before movement over the contralateral SMA could be a compensatory mechanism stronger in Parkinson’s disease without FOG and Parkinson’s disease with mild FOG. On the other hand, early high-beta and interrupted low-beta ERD along with excessive theta ERS over Cz and Fc1 are exclusive EEG features of Parkinson’s disease with severe FOG. This again suggests a different underlying mechanism for mild and severe FOG and possibly different FOG subtypes (e.g. motor, cognitive, limbic).

Low- and high-beta activities are involved in motor control at both cortical and subcortical regions. Modulation of beta activities, in general, has been introduced to play a role in internal timing, especially putaminal beta.^62^^,^^63^ Distinct projections from (pre-)SMA to different parts of putamen suggest that the striatum tracks regulate movement via modulation of beta activity.^64^ The power of the beta-band reflects the level of motor preparation, and beta ERS has been shown to contribute to movement inhibition.^65^^,^^66^ Plus, different beta frequency bands seem to have different distributions and functionalities in different brain structures. Cortical low-beta activities are related to the speed of the movement,^67^ and the maximum low-beta coherence was highest in the lateral M1 region,^37^ which corresponds to the hand areas of the motor cortex and is responsive to dopaminergic treatments.^68^ In contrast, cortical and STN high-beta frequency band activities have been reported to be related to self-paced movement and are less affected by dopamine.^68^^,^^69^ The maximal coherence in the high-beta activity was reported in the midline cortex corresponding to the SMA, cingulate cortex and leg area of M1.^37^ The frequency modulation patterns of the low-beta band have been shown to be more sensitive to dopaminergic treatments compared with high-beta,^70^ which suggests the role of high-beta band frequency modulation in FOG, and the importance of frequency modulation of brain oscillations as a communication tool in the brain along with power modulation. It has recently been introduced that failure of cortical–subthalamic frequency modulation information processing and communication in Parkinson’s disease can result in FOG.^71^ In addition, the association of NS1 and low-beta ERD over Cz with FOG severity during movement preparation suggests a possible relationship between these two EEG features.

The presence of excessive theta ERS between two auditory cues (especially over M1) changes relative to the severity of FOG with the highest and longest synchrony in Parkinson’s disease with severe FOG over M1 followed by both high-beta and low-beta ERD. The presence of excessive theta ERS, associated with FOG, is consistent with the previous study.^35^ This might suggest a different mechanism that controls voluntary movements in Parkinson’s disease with FOG, particularly in severe cases. Theta activities are related to the cognitive function of the brain, motor sequence learning and stabilization.^72^ Excessive theta ERS indicates impairment of motor circuitry related to already learned movements and a relationship between the cognitive and attention impairment as well as FOG severity. Higher cognitive load of gait compared to sitting and standing could be one of the factors that contribute to FOG occurrence.

The relationship between MRCP, brain oscillations and EMG activities is not yet completely known; however, different parts of the unified concept of motor training,^73^ integrated investigations of the cortical signals in different FOG subtypes and severities can shed light on underlying mechanisms of FOG and motor control in the human brain. The relationship between MRCP and FOG severities has never been investigated before. The results from this study emphasize the crucial involvement of the motor cortex in the complex pathophysiology of FOG. Further research including experiments with self-initiated, right and left, upper and lower limb movements, and motor imagery tasks in different FOG subtypes and severities can help clarify the role of the changes in MRCP characteristics as well as brain oscillations in FOG, and in human brain in general. Based on the results from this study, the abnormalities in different motor cortical areas might be used as FOG detection, and biomarkers of severe cases as particular subtypes of FOG might be more prone to severe FOG. Furthermore, impairment of motor cortical areas can offer alternative treatment options for FOG based on TMS and tDCS on motor cortical regions, which has been shown to be effective.^74^ BCI-based rehabilitation systems are also emerging and promising technologies that use cortical information to rehabilitate FOG by assistive devices. Motor cortical activities are essential missing parts of the FOG dilemma, and investigation of FOG-associated malfunctions of these parts can help uncover the underlying mechanism of FOG as well as providing new treatment options.

## Supplementary material

Supplementary material is available at *Brain Communications* online.

## Supplementary Material

fcab277_Supplementary_Data

## Abbreviations

ADF =: ankle dorsiflexion
BCI =: brain–computer interface
BG =: basal ganglia
BP =: Bereitschaftspotential
CNV =: contingent negative variation
DBS =: deep brain stimulation
ERD/ERS =: event-related (de)synchronization
FOG =: freezing of gait
ICA =: independent component analysis
IMU =: inertial measurement unit
LED =: levodopa equivalent dose
MRCP =: movement-related cortical potential
NS =: negative slope
SMA =: supplementary motor area
SOL =: soleus muscles
STN =: subthalamic nucleus
TA =: tibialis anterior
tDCS: transcranial direct current stimulation
TMS =: transcranial magnetic stimulation
TKEO =: Teager–Kaiser Energy Operator
UPDRS =: Unified Parkinson’s Disease Rating Scale
