# Supplementary material for: Movement-related EEG signatures associated with freezing of gait in Parkinson’s disease: an integrative analysis
Source: Brain Commun. 2021 Nov 24;3(4):fcab277. doi: 10.1093/braincomms/fcab277 (PMC8643573; doi:10.1093/braincomms/fcab277)
Supplement: fcab277_Supplementary_Data [file fcab277_supplementary_data.pdf]

Supplementary Materials for:

"Movement Related EEG Signatures Associated with Freezing of Gait in Parkinson's Disease: An Integrative Analysis "

(Short Running Title: Freezing of Gait and Motor Cortical EEG)

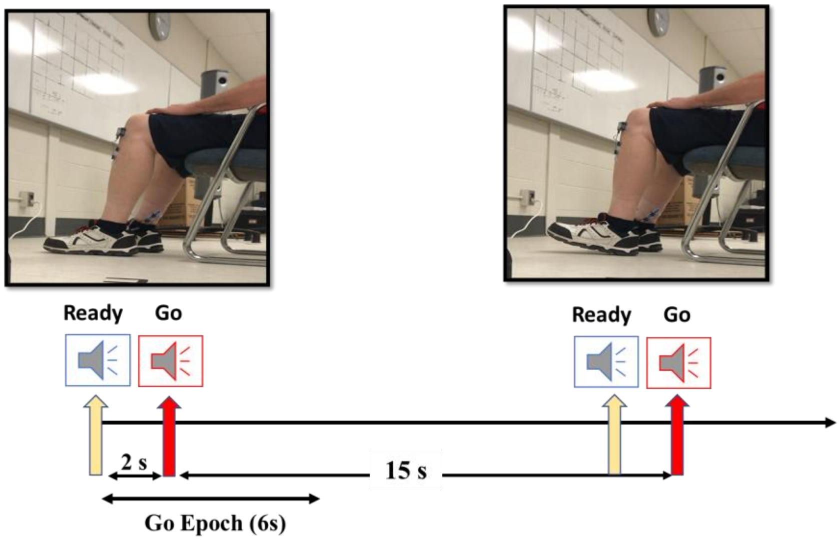

Supplementary figure 1: Time course of the auditory cues and ‘Go’ epoch for one trial of the experimental protocol.

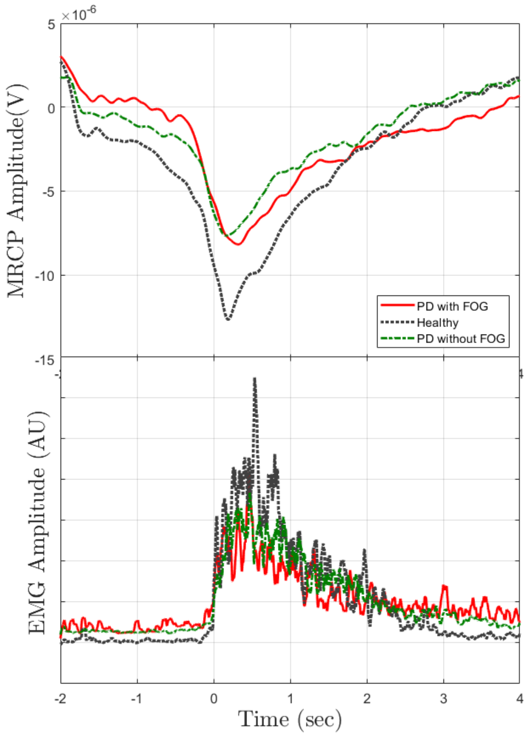

Supplementary figure 2: Averaged MRCP over ‘Go’ epochs from healthy controls and PD groups from Cz channels and the corresponding average EMG-TA. In each plot, the black dotted line, the green dashed line, the red solid line, the light blue solid line, and dark blue solid line represent the epoch average for healthy control, PD patients without FOG, and PD patients with FOG groups, respectively.

Table 1 Ages for all participants and UPDRS scores, duration of disease, and LED for all PD patients in each group: H stands for healthy controls group, F stands for PD patients with FOG group, NF stands for PD patients without FOG group.

**Participant type**	**code**	**Gender (Male/Female)**	**Age**	**duration of disease (year)**	**UPDRS-III**	**LED**
**PD without FOG**	1 *	M	77	6	42	100
	2	M	75	1	22	0
	3	F	87	5	37	400
	4	M	68	6	26	100
	5	F	74	7	36	100
	6	M	84	15	22	368
	7	M	65	13	18	1100
	8	F	68	4	35	700
	9	M	80	6	21	325
	10	M	75	17	35	600
	11	M	77	7	30	1000
	12	M	69	17	17	799
	13	M	66	5	29	100
	14 *	M	78	4	31	600
**PD with FOG** m: mild FOG s: severe FOG	1	M	89	4	49	100
	2 *	M /m	76	7	17	550
	3	M /m	80	21	38	600
	4	M / s	81	16	25	1520
	5	M /m	78	15	37	701
	6	M / s	80	8	25	1100
	7	M	63	15	13.5	900
	8	F /m	68	6	34	600
	9 *	M /s	79	3	20	1000
	10	M /s	80	4	33	1000
	11	M /m	90	7	58	100
	12	M /m	77	7	31	1000
	13	M /m	68	14	25	550
	14	M /s	78	8	37	225
**Healthy Controls**	1	M	83	N/A	N/A	N/A
	2 *	M	76	N/A	N/A	N/A
	3	M	77	N/A	N/A	N/A
	4	F	83	N/A	N/A	N/A
	5	M	68	N/A	N/A	N/A
	6	M	79	N/A	N/A	N/A
	7	M	68	N/A	N/A	N/A
	8	F	72	N/A	N/A	N/A
	9	M	80	N/A	N/A	N/A
	10	M	78	N/A	N/A	N/A
	11	M	89	N/A	N/A	N/A
	12	F	78	N/A	N/A	N/A
	13	M	78	N/A	N/A	N/A

*** Dominant foot of subjects with * is the left foot.**

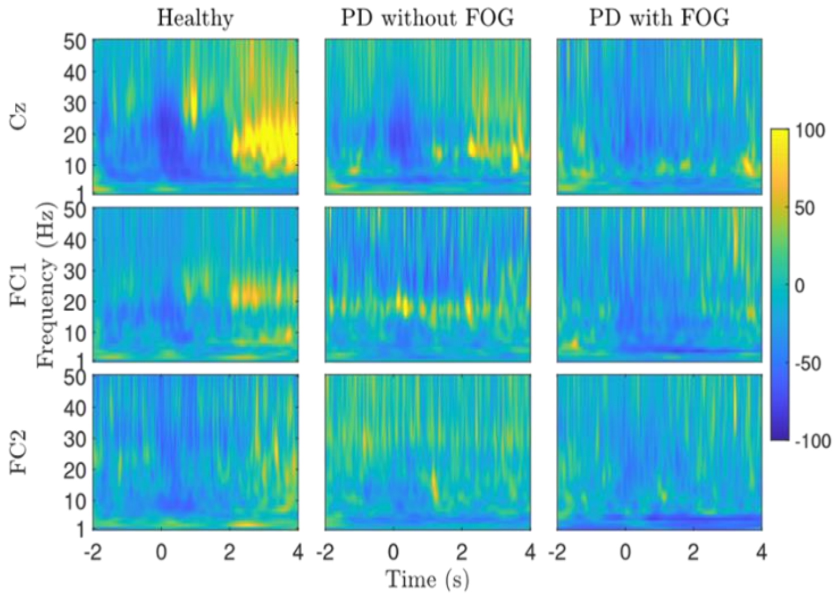

(a) Time frequency representation of ERD/ERS

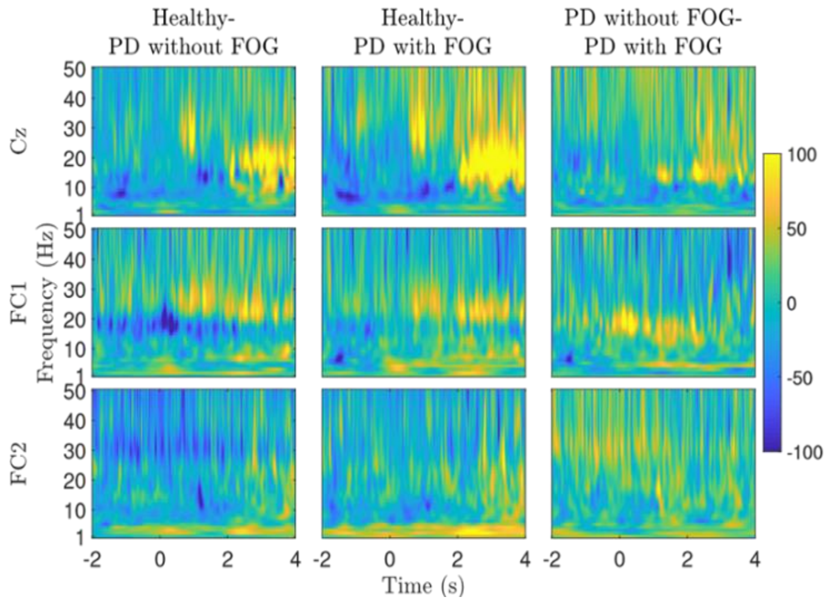

(b) Time frequency representation of ERD/ERS difference

Supplementary figure 3: Time-frequency representations of ERD/ERS and ERD/ERS differences in three channels (Cz, FC1, FC2) of three groups: Healthy controls, PD patients without FOG, and PD patients with FOG. In plot (a), ERD/ERS indicating percentage change relative to baseline of -4 s to -2 s are represented as blue/yellow colors, respectively between 1 Hz and 50 Hz from -2 s to 4 s. In plot (b), time-frequency representations of ERD/ERS differences in three channels (Cz, FC1, FC2) among three groups, indicated as ’Healthy - PD without FOG’, ’Healthy - PD with FOG’, ’PD without FOG - PD with FOG’.

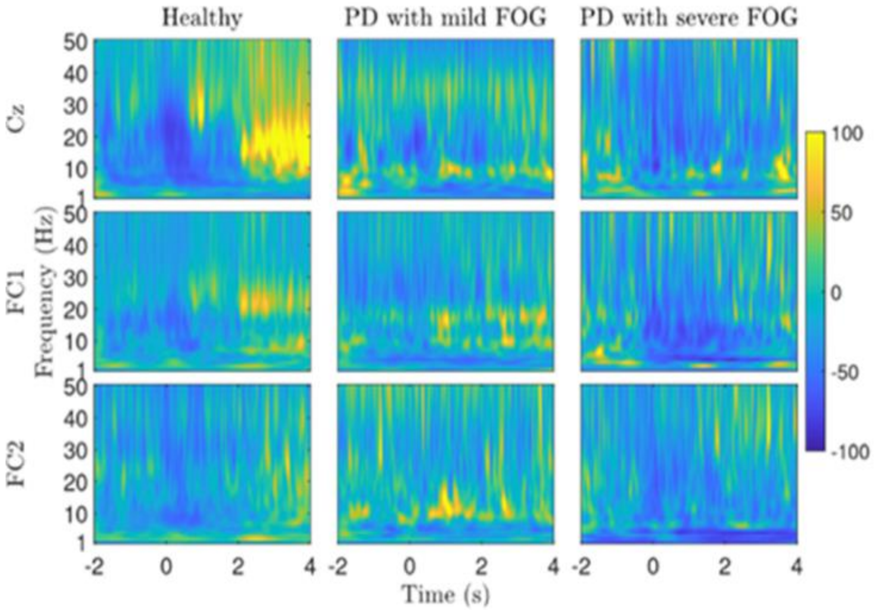

(a) Time frequency representation of ERD/ERS

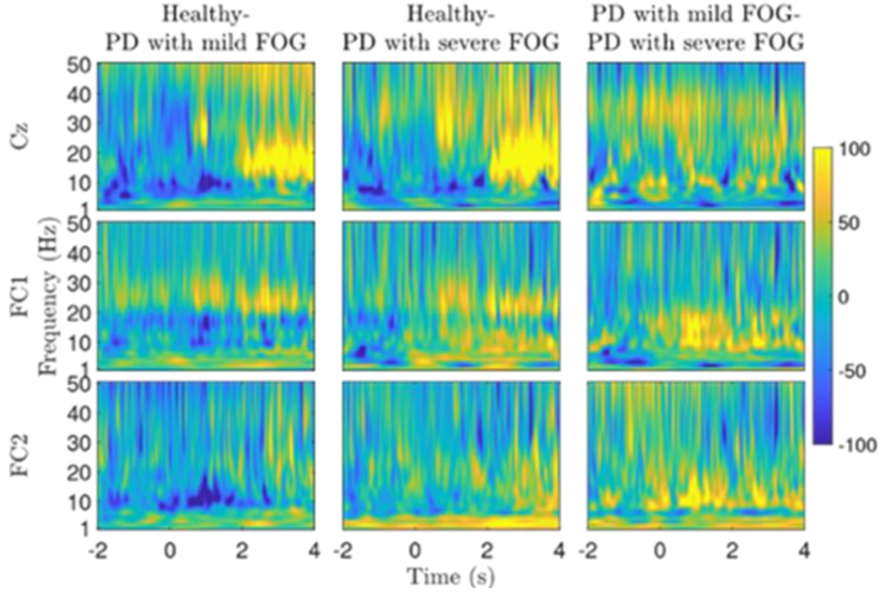

(**b**) Time frequency representation of ERD/ERS difference

Supplementary figure 4: Time-frequency representations of ERD/ERS in three channels (Cz, FC1, FC2) of three groups: Healthy controls, PD patients with mild FOG, and PD patients with severe FOG. In plot (a), ERD/ ERS indicating percentage change relative to a baseline of -4 s to -2 s are represented as blue/yellow colors, respectively between 1 and 50 Hz from -2 s to 4 s. In plot (c), time-frequency representations of ERD/ERS differences in three channels (Cz, FC1, FC2) among three groups, indicated as ’Healthy - PD with mild FOG’, ’Healthy - PD with severe FOG’, ’PD with mild FOG - PD with severe FOG’.
